# tsRNA-Ala-3–0030 drives ovarian cancer progression by suppressing ZNF70

**DOI:** 10.3389/fonc.2026.1738006

**Published:** 2026-01-29

**Authors:** Xinchen Wang, Ying Zhou, Xinhao Zhou, Bing Zhang, Hongwei Liang, Youguo Chen

**Affiliations:** 1 Department of Obstetrics and Gynecology, The First Affiliated Hospital of Soochow University, Suzhou, China; 2 Department of Obstetrics and Gynecology, Tongde Hospital of Zhejiang Province, Hangzhou, China; 3 Suzhou Key Lab of Multi-modal Data Fusion and Intelligent Healthcare, Suzhou, China; 4 University of Edinburgh, Edinburgh, United Kingdom; 5 Wuxi Maternal and Child Health Care Hospital, The Affiliated Women’s Hospital of Jiangnan University, Wuxi, China; 6 Department of Life Sciences and Technology, China Pharmaceutical University, Jiangsu, China

**Keywords:** ovarian cancer, small non coding RNA, tsRNA, tsRNA-Ala-3-0030, ZNF70

## Abstract

Globally, ovarian cancer remains a leading cause of gynecologic cancer death. This poor prognosis is primarily attributed to advanced disease at diagnosis, a high propensity for recurrence, and the inadequate therapeutic efficacy of current standard regimens. Emerging evidence has established the functional significance of transfer RNA-derived small RNAs (tsRNAs) in tumor biology. Nevertheless, their exact mechanisms and roles in ovarian cancer pathogenesis are not yet fully understood. Our research was designed to profile tsRNA expression, verify its presence in ovarian cancer tissues and established cell lines, and leverage cellular and animal models to decipher the functional roles and molecular mechanisms of these molecules. Our analysis revealed that tsRNA-Ala-3–0030 is significantly upregulated in ovarian cancer models. Higher expression levels of this tsRNA were associated with enhanced malignant behaviors, including proliferation, migration, and invasion. The suppression of tsRNA-Ala-3–0030 expression attenuated these malignant phenotypes, whereas its overexpression promoted tumor progression in both cellular cultures and xenograft models. Mechanistically, tsRNA-Ala-3–0030 directly targeted the tumor suppressor ZNF70, leading to its downregulation and consequent promotion of ovarian cancer growth and metastasis. Restoration of ZNF70 expression reversed the oncogenic effects of tsRNA-Ala-3-0030, confirming its pivotal role in mediating this pathway. Collectively, our findings reveal tsRNA-Ala-3–0030 as a previously unrecognized oncogenic regulator that promotes ovarian cancer progression through ZNF70 downregulation. This study elucidates a tsRNA-dependent tumorigenic mechanism and positions tsRNA-Ala-3–0030 as a dual-purpose prognostic indicator and therapeutic target, advancing personalized treatment strategies.

## Introduction

Ovarian cancer’s aggressive biology and propensity for late-stage diagnosis together maintain its standing as a major cause of cancer death in women globally (1). The characteristically indolent early presentation of ovarian cancer contributes to its prevalent late-stage detection, which in turn sustains a poor clinical outcome with only incremental improvements attributed to current therapeutic strategies (1). Worldwide, ovarian cancer represents the eighth most common cancer in women, accounting for approximately 3.7% of incident cancer cases and 4.7% of cancer-associated deaths in 2020 (2). The cornerstone of ovarian cancer management integrates cytoreductive surgery with platinum-taxane doublet chemotherapy. However, a high incidence of disease recurrence commonly follows this therapeutic approach, constituting a major factor underlying adverse long-term survival (3). Ovarian cancer pathogenesis is driven by a combination of genetic alterations—including mutations in oncogenes like *KRAS* and tumor suppressor genes such as *BRCA1/2*—alongside dysregulated epigenetic mechanisms (4). However, the molecular mechanisms that drive tumor initiation and progression remain incompletely understood. For the first time, research has systematically delineated the evolving miRNA landscape throughout the malignant transition from endometriosis to ovarian carcinoma. This work establishes that increasing expression of critical miRNAs, particularly within the miR-200 family, correlates strongly with tumor development (5). The findings offer a new molecular framework for developing early diagnostic biomarkers and targeted therapies, reinforcing the necessity to innovate both biomarkers and therapeutic agents to advance personalized treatment strategies (6).

tsRNAs are a newly recognized category of sncRNAs, defined by their length of approximately 15 to 40 nucleotides (7). Rather than arising from stochastic degradation, tsRNAs originate from highly controlled processing of tRNA precursors or mature tRNAs at specific cleavage sites (7). The specific cleavage sites determine the classification of tsRNAs into two subtypes: tRNA-derived fragments (tRFs) and tRNA halves (tiRNAs) (7). Depending on retention of the 5′ or 3′ anticodon loop sequence, tiRNAs are categorized as either 5′-tiRNAs or 3′-tiRNAs (8, 9). Originating from both precursor and mature tRNAs, tRFs are categorized according to cleavage location into tRF-1, tRF-3, tRF-5, tRF-2, and i-tRF subtypes (10). Beyond their biogenesis, tsRNAs exhibit diverse biological functions. tsRNAs execute their functions via mRNA binding or protein interactions, thereby regulating signal transduction pathways, controlling gene expression, modulating cell cycle progression, and influencing epigenetic regulation and chromatin modifications (7). Accumulating data associate tsRNAs with various cancer types (11), the exact mechanistic contributions of tsRNAs to ovarian cancer biology are still largely undefined.

In this study, we identified tsRNA-Ala-3–0030 as a previously uncharacterized tsRNA significantly upregulated in ovarian cancer. Through a combination of bioinformatics, molecular assays, and functional experiments *in vitro* and *in vivo*, we investigated its oncogenic properties and underlying mechanism. Our work establishes that tsRNA-Ala-3–0030 promotes ovarian tumorigenesis through direct inhibition of ZNF70. This reveals novel tsRNA functions in ovarian cancer and positions tsRNA-Ala-3–0030 as a dual-purpose candidate biomarker and therapeutic target.

## Materials and methods

### Sample collection

Clinical ovarian cancer samples were acquired from patients managed at the Department of Obstetrics and Gynecology, First Affiliated Hospital of Soochow University (Ethics Approval No. 027(2021)). Fresh tumor tissues were processed in two ways: fixation in paraformaldehyde for immunohistochemistry or rapid freezing in liquid nitrogen for RNA extraction. Clinical data were collected from 24 patients diagnosed with ovarian cancer and 8 healthy ovarian tissue specimens. (All diagnoses were confirmed according to current International Federation of Gynecology and Obstetrics (FIGO) staging criteria.), as detailed in **Supplementary Table S1**.

### Cell culture and small RNA transfection

HEY cells were acquired from Wuhan Pricella Biotechnology Co., Ltd. (China). The HO8910 and IOSE80 cell lines were generously provided by the Department of Obstetrics and Gynecology at the First Affiliated Hospital of Soochow University. Cells were grown in their respective media: HEY in High-glucose DMEM, and HO8910 and IOSE80 in RPMI-1640. All media were supplemented with 10% FBS (TransGen Biotech, China), and cells were incubated under standard conditions (37°C, 5% CO_2_).

tsRNA mimics, inhibitors, agomirs, and antagomirs were obtained from RiboBio (Guangzhou, China), with sequences listed in **Supplementary Table S3**. Small interfering RNAs (100 nM) targeting human ZNF70 were purchased from Biotend (Shanghai, China), as detailed in **Supplementary Table S4**. Plasmids were supplied by Genomeditech (Shanghai, China). Cell transfection was carried out employing Lipofectamine 3000 (Invitrogen, USA) as per the vendor’s protocol.

### Analysis of gene expression by quantitative RT-PCR

Total RNA was isolated from cells using TRIzol reagent (Vazyme, China). According to the manufacturer’s protocol, cDNA was synthesized from RNA samples using a stem-loop method-based miRNA 1st Strand cDNA Synthesis Kit (Vazyme, China). Quantitative reverse transcription PCR was performed in triplicate with SYBR Green master mix (TransGen Biotech, China) using a LightCycler^®^ 96 platform (Roche). The amplicon size for tsRNA-Ala-3–0030 is 68 bp and the PCR amplification was carried out for 50 cycles. The primer sequences used are listed in **Supplementary Table S5**.

### Cell viability, migration, and invasion assays

To assess cell viability, the CCK-8 assay (Life-iLab, China) was employed. Hey cells were seeded at a density of 3× 10³ cells per well in 96-well plates. To evaluate proliferative activity, the EdU DNA Proliferation Kit (Beyotime Biotechnology, China) was used according to the supplied protocol. Hey cells were seeded at a density of 3× 10³ cells per well in 96-well plates. Fluorescence images of EdU-positive cells were acquired with an Olympus microscope. Cell invasion was evaluated by transwell chambers pre-coated with Matrigel (Corning, USA). To assess migration, after Hey cells reaching 70–80% confluence, cell concentration was determined and adjusted to 2.5 × 10^5^ cells/mL and were suspended in serum-free medium and added to the 24-well transwell upper insert (8-μm pore size, Corning, USA), with the lower compartment filled with High-glucose DMEM containing 10% FBS serving as a chemoattractant. Following a 48-hour incubation, the membranes were fixed and subsequently stained with 0.1% crystal violet. The stained cells were then imaged under an Olympus microscope. Cell migratory ability was determined using a wound healing assay. HEY cells were plated in 6-well plates (2.5×10^5^ cells/well) and incubated for 24 hours to reach full confluence. To initiate the wound healing assay, a uniform scratch was made with a sterile 200-μL pipette tip and the serum concentration was adjusted to 1% FBS. Cell migration into the wound area was monitored at 0 and 24-hour time points under a phase-contrast microscope. Quantification of the wound healing distance was performed with ImageJ software.

### Western blot analysis and immunohistochemistry

Cellular proteins were harvested in RIPA buffer, separated via SDS-PAGE, and were electrophoretically transferred to PVDF membranes. To reduce background, the membranes were treated with 5% BSA for 1 hour at room temperature. Subsequently, they were incubated with an anti-ZNF70 primary antibody (Invitrogen, USA) and GAPDH (ABclonal, China) at and 4°C overnight. For detection, the washed membranes were incubated with HRP-conjugated secondary antibodies (1 h, room temperature) (ABclonal, China). Blots were developed using working solution prepared by mixing ECL reagent (Affinity, China) and NcmECL Ultra (New Cell & Molecular Biotech, China) according to the manufacturer’s instructions, followed by imaging with a Tanon Science & Technology Co., Ltd. (Shanghai, China). For IHC, tumor tissues were fixed, sectioned into 5-μm slices, and stained for ZNF70 using standard immunohistochemistry protocols.

### RNA fluorescence *in situ* hybridization

5′FAM-labeled tsRNA-Ala-3–0030 probes were synthesized by BersinBio (Guangzhou, China). HEY cells were cultured on 20-mm confocal dishes and transfected with tsRNA mimics, tsRNA inhibitors, or corresponding controls. For subsequent staining, 48-hour cultured cells were subjected to fixation in 4% paraformaldehyde (20 min), followed by permeabilization using 0.5% Triton X-100 (10 min) and a second fixation step with 1% paraformaldehyde (10 min). After being subjected to graded ethanol dehydration, samples were incubated with the probe mixture (1.25 pmol/μL) for overnight hybridization at 42°C. Following washes, nuclear counterstaining was carried out with DAPI, and fluorescence images were acquired using confocal microscopy.

### Luciferase reporter assay

Cloning of the ZNF70 3′UTR into the pmirGLO-basic vector yielded the luciferase reporter construct. Co-transfection assays were performed in HEY cells using tsRNA-Ala-3–0030 mimics/controls in combination with wild-type or mutant ZNF70 3’UTR reporter plasmids. To determine the effect, the relative firefly luciferase activity was quantified 48 hours post-transfection with a Dual-Luciferase Reporter Assay Kit (Promega, USA) on a FLUOstar Omega system (BMG LABTECH, Germany), with Renilla luciferase serving as the normalization control.

### Tumor xenograft assay

All animal experiments utilized female BALB/c nude mice (nu/nu, 5–6 weeks old) supplied by Gem Pharmatech (China). The Institutional Animal Care and Use Committee (IACUC) of Soochow University reviewed and approved the animal study protocol. For subcutaneous tumor formation, a suspension of HEY cells (4×10^6^) in 50 μL of 1:1 diluted Matrigel was prepared and administered into the dorsal flank region of mice. When tumors reached approximately 100 mm³, mice were randomized into four groups (n = 5 per group) and treated intratumorally every 3 days with agomir-NC, agomir (5 nmol per injection, RiboBio), antagomir-NC, or antagomir (20 nmol per injection, RiboBio). Following four treatment cycles, mice were euthanized and tumors were excised for subsequent volume and tumor weight measurements.

### Public data and bioinformatics analysis

The RNA-sequencing and clinical data for ovarian cancer patients (TCGA-OV) were downloaded from the TCGA portal (https://portal.gdc.cancer.gov/) and normalized using TPM. Based on median gene expression, patients were categorized into high- and low-expression groups, and survival analysis was implemented with the survival and survminer packages in R, as detailed in **Supplementary Table S2**. The tsRNA-Ala-3–0030 sequence (tRF-24-7SHRJFWRE2) was retrieved from MINTbase. Potential target genes were predicted using miRanda, TargetScan, and RNAhybrid. To identify differentially expressed genes, we employed the LIMMA package for statistical analysis. Hallmark gene sets were downloaded from MSigDB, and pathway enrichment analysis was conducted using clusterProfiler (v4.12.6), with a FDR P-value of less than 0.05 considered statistically significant.

### Statistical analysis

All experiments were independently repeated three times, with data presented as mean ± SD. For comparisons between two groups, a two-tailed Student’s t-test was applied, whereas the Wilcoxon rank-sum test was used for tumor versus normal tissue expression analysis. Kaplan-Meier survival curves were constructed, and intergroup comparisons were evaluated via log-rank testing, adopting a significance threshold of P < 0.05.

## Results

### tsRNA-Ala-3–0030 is highly expressed in ovarian cancer

**Figure 1:**
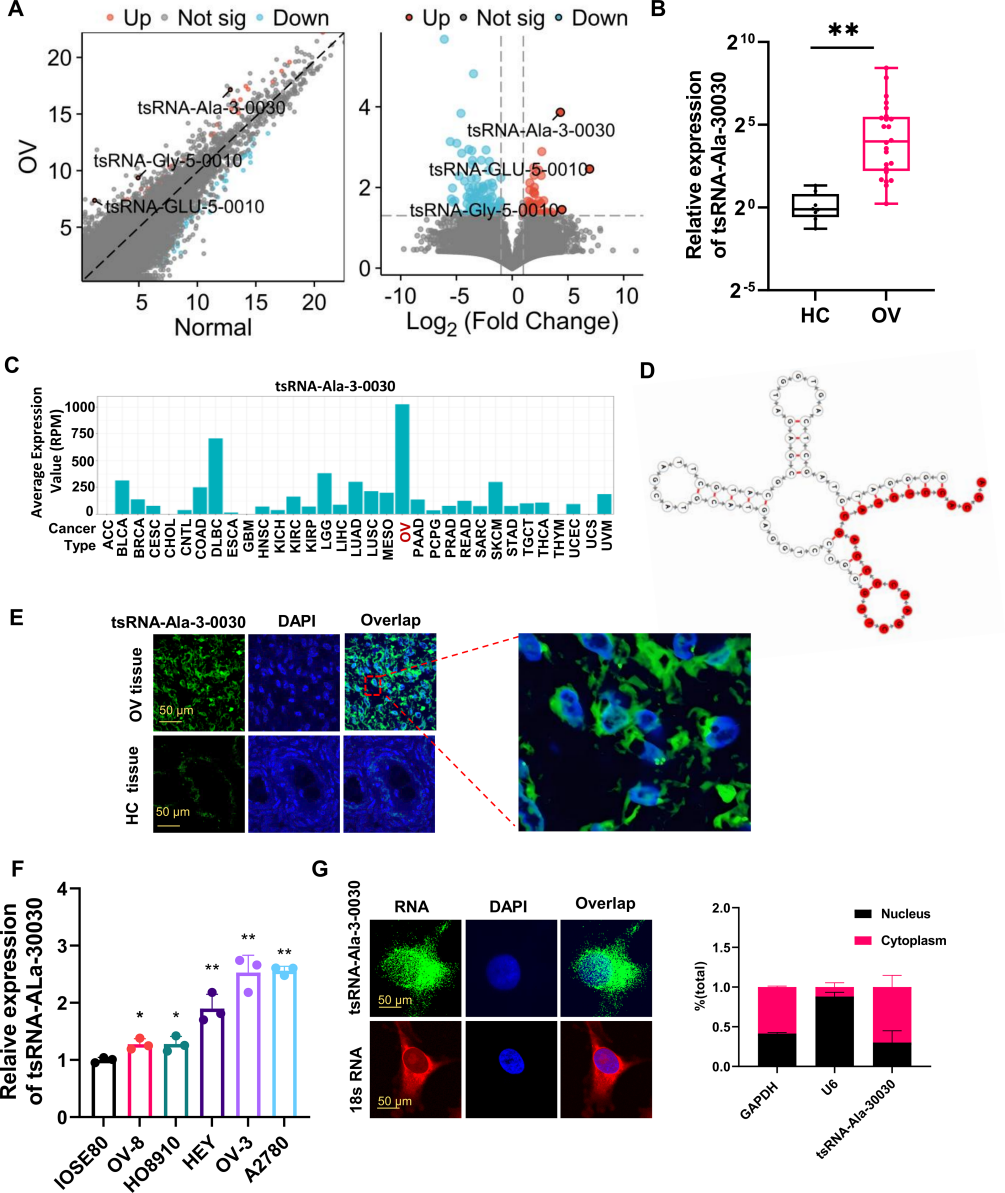
tsRNA-Ala-3–0030 is highly expressed in ovarian cancer. **(A)** Identification of the top three dysregulated tsRNAs in ovarian cancer versus normal tissues. A volcano plot was generated based on data sourced from the tsRFun database. **(B)** qPCR analysis of the expression of tsRNA-Ala-3–0030 in ovarian cancer tissues. **(C)** The average expression value of tsRNA-Ala-3-0030 in multiple cancers according to the tRFfinder pan-cancer database. **(D)** The secondary structure of tRNA-Ala that was the origin of tsRNA-Ala-3-0030. **(E)** FISH assay for detecting the expression of tsRNA-Ala-3–0030 in ovarian cancer tissues. **(F)** The figure depicts the expression profile of tsRNA-Ala-3-0030, as measured by quantitative real-time PCR (qRT-PCR), in various ovarian cancer cell lines. **(G)** FISH assay for detecting the location of tsRNA-Ala-3–0030 in HEY cells (GAPDH was used as the endogenous reference gene for normalizing mRNA expression levels and U6 snRNA was used as the endogenous reference for normalizing small non-coding RNA expression levels in qRT-PCR assays. 18S rRNA was used as the loading control). *P < 0.05, **P < 0.01.

To decipher tsRNA functions in ovarian tumorigenesis, we conducted tsRNA expression profiling through the tsRFun database platform (8). Three tsRNAs—tsRNA-Ala-3-0030, tsRNA-Gly-5-0010, and tsRNA-Glu-5-0010—were significantly upregulated in ovarian cancer tissues (**Figure 1A**). These candidates were further validated by qPCR in six paired ovarian cancer and adjacent normal tissues. Both tsRNA-Ala-3–0030 and tsRNA-Gly-5–0010 were notably overexpressed in tumor samples (**Figure 1B**; **Supplementary Figure S1A**), while tsRNA-Glu-5–0010 showed no significant difference between ovarian cancer and adjacent normal tissues (**Supplementary Figure S1B**). Additionally, data from the tRFinder pan-cancer database revealed specific enrichment of tsRNA-Ala-3–0030 in ovarian cancer (**Figure 1C**). Thus, tsRNA-Ala-3–0030 was selected for further study. tsRNA-Ala-3-0030’s secondary structure formed a typical cloverleaf pattern, yielding the functional 24-nucleotide fragment through specific processing (**Figure 1D**). Fluorescence *in situ* hybridization (FISH) confirmed the elevated expression of tsRNA-Ala-3–0030 in ovarian cancer tissues, where it was predominantly localized in the cytoplasm (**Figure 1E**). Furthermore, tsRNA-Ala-3–0030 demonstrated elevated expression in ovarian cancer cells and was predominantly detected in the cytoplasmic compartment (**Figures 1F, G**).

### tsRNA-Ala-3–0030 promote ovarian cancer cell progression

**Figure 2:**
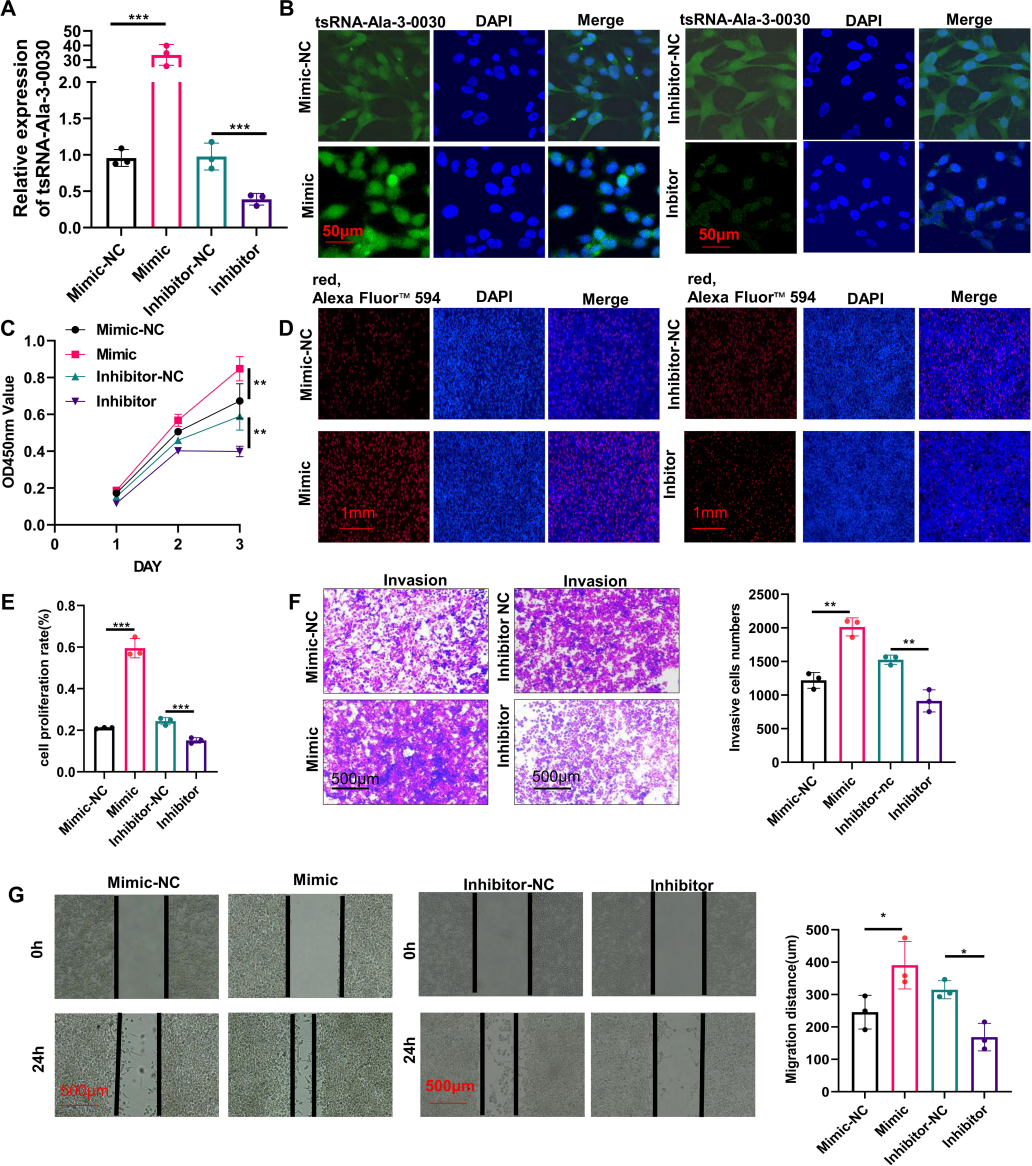
tsRNA-Ala-3–0030 promote ovarian cancer cell progression *in vitro*. **(A, B)** FISH and qPCR assay for detecting the expression of tsRNA-Ala-3–0030 in HEY cells transfected with tsRNA-Ala-3–0030 inhibitor or mimic. **(C)** CCK8 analysis for the growth of HEY cells transfected with tsRNA-Ala-3–0030 inhibitor or mimic. **(D, E)** EdU incorporation assay was performed to evaluate the proliferative capacity of HEY cells upon transfection with tsRNA-Ala-3–0030 inhibitor or mimic. **(F)** Transwell assays for measuring the invasion of HEY cells transfected with tsRNA-Ala-3–0030 inhibitor or mimic. **(G)** A wound healing assay was performed to evaluate the migration of HEY cells. *P < 0.05, **P < 0.01, ***P < 0.001.

Inhibition of tsRNA-Ala-3–0030 significantly suppressed its expression, while mimic administration resulted in pronounced overexpression (**Figures 2A, B**). As shown in **Figures 2C–E**, the tsRNA-Ala-3–0030 inhibitor markedly reduced cell proliferation, whereas the mimic promoted cell proliferation, as assessed by the CCK-8 (**Figure 2C**) and EdU assays (**Figures 2D, E**). Transwell and wound healing analyses confirmed that tsRNA-Ala-3–0030 inhibition significantly impaired cellular invasion (**Figure 2F**) and migration (**Figure 2G**), whereas its overexpression markedly enhanced migratory (**Figure 2F**) and invasive (**Figure 2G**) capacities.

**Figure 3:**
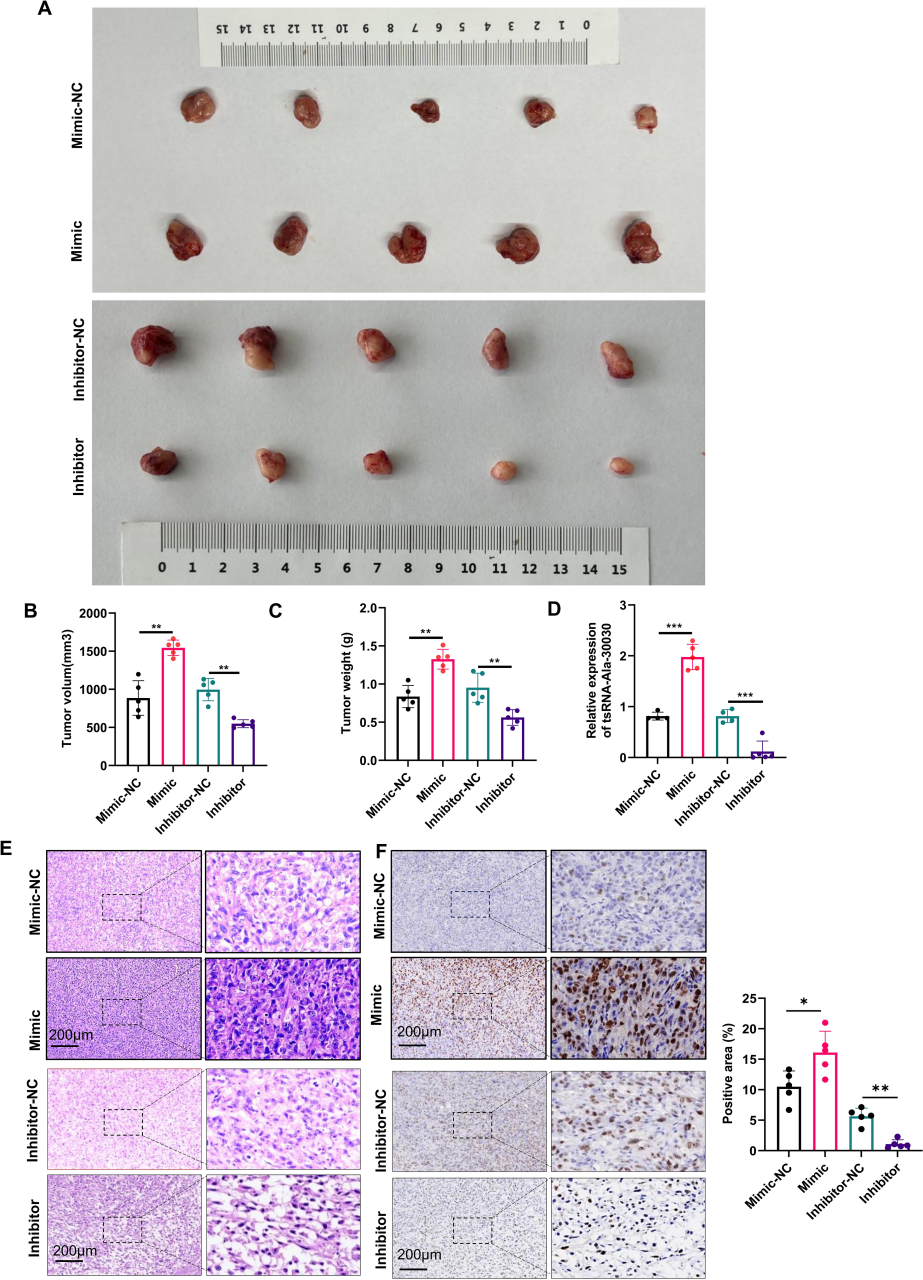
tsRNA-Ala-3–0030 promote ovarian cancer cell progression *in vivo*. **(A)** Representative tumor images show the *in vivo* effect of tsRNA-Ala-3–0030 inhibitor or mimic in a xenograft mouse model. **(B, C)** Quantification of tumor volume and weight in xenograft models following treatment with tsRNA-Ala-3–0030 inhibitor or mimic. **(D)** Analysis of tsRNA-Ala-3–0030 expression by qPCR in tumor tissues from xenograft mice treated with tsRNA-Ala-3–0030 inhibitor or mimic. **(E, F)** Histological analysis by H&E and Ki-67 staining was performed on tumor tissues from xenograft nude mice treated with tsRNA-Ala-3-0030. *P < 0.05, **P < 0.01, ***P < 0.001.

An *in vivo* xenograft model was created in nude mice using HEY cells to investigate tsRNA-Ala-3-0030’s biological role. Treatment involved every three days administration of tsRNA-Ala-3–0030 inhibitor or mimic. No significant differences in body weight were observed among the experimental cohorts. However, treatment with the tsRNA-Ala-3–0030 inhibitor reduced tumor growth, while the mimic promoted tumor growth (**Figures 3A-E**). Additionally, the tsRNA-Ala-3–0030 inhibitor decreased Ki67 expression in tumor tissues, whereas the mimic increased Ki67 expression (**Figure 3F**; **Supplementary Figure S1C**). H&E staining demonstrated reduced tumor cellularity and expanded necrotic regions in the tsRNA-Ala-3–0030 inhibitor group relative to controls (**Figure 3E**), suggesting an inhibition of tumor aggressiveness. Ki67 immunohistochemical quantification revealed significantly elevated proportions of Ki67-positive cells in tumors overexpressing tsRNA-Ala-3-0030 (**Figure 3F**), confirming its role in markedly promoting tumor cell proliferation *in vivo*.

### ZNF70 is a direct target of tsRNA-Ala-3-0030

**Figure 4:**
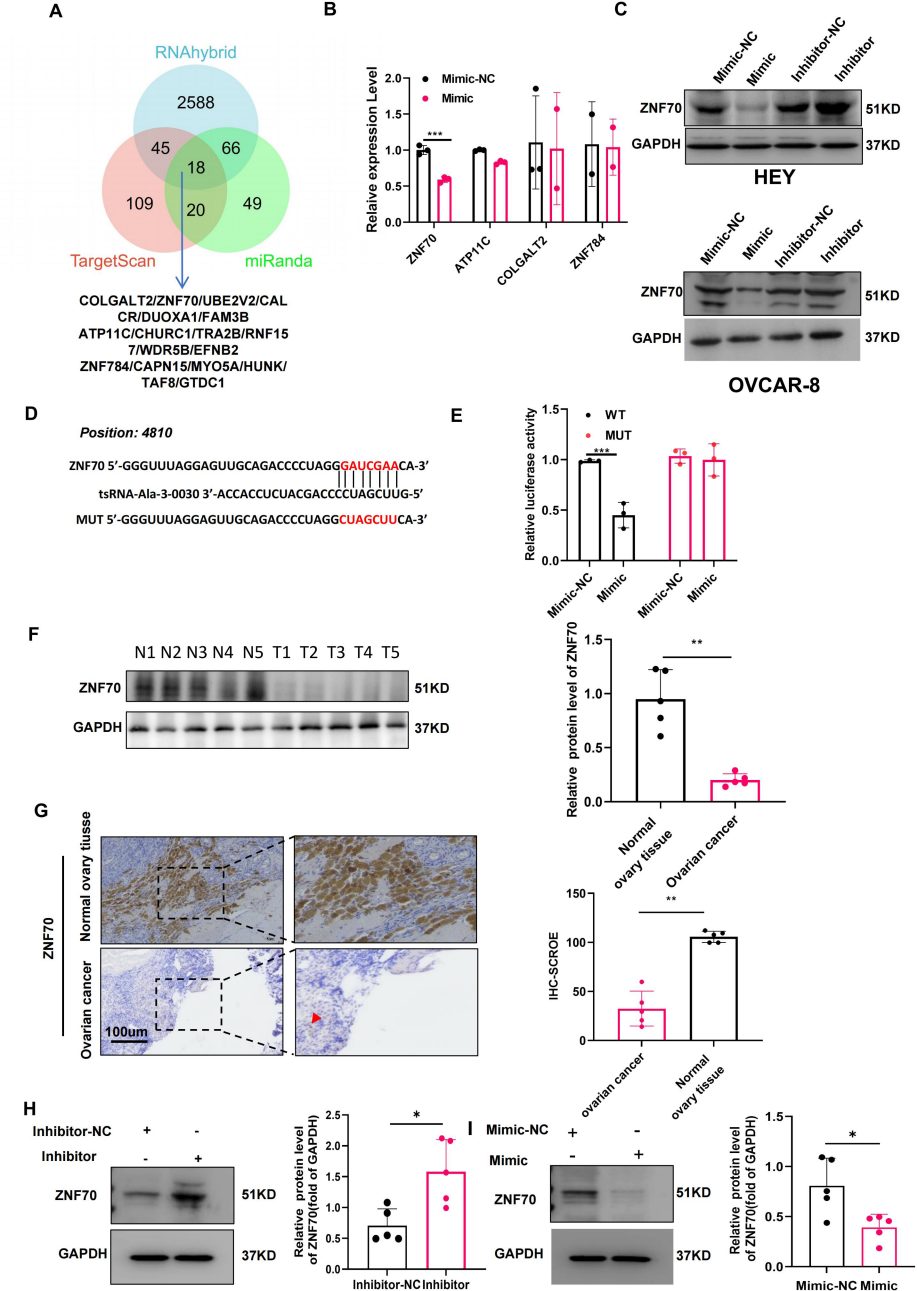
ZNF70 is a direct target of tsRNA-ALa-3-0030. **(A)** Identification of potential target genes of tsRNA-Ala-3–0030 based on miRanda, TargetScan, and RNAhybrid. **(B)** Analysis of ZNF70, ATP11C, COLGALT2, and ZNF784 expression by qPCR in HEY cells transfected with tsRNA-Ala-3–0030 mimic or negative control. **(C)** Analysis of ZNF70 expression by Western blot in HEY and OVCAR-8 cells transfected with tsRNA-Ala-3–0030 mimic, inhibitor, or negative control (NC). **(D)** The predicted binding site between ZNF70 and tsRNA-Ala-3-0030. **(E)** The luciferase activity in tsRNA-Ala-3–0030 mimic-transfected HEY cells that co-transfected with WT or MUT luciferase plasmid. **(F, G)** Western blotting and immunohistochemistry were performed to detect ZNF70 expression levels in ovarian cancer tissues and their normal counterparts. **(H)** Analysis of ZNF70 expression by Western blot in xenograft tumors from mice treated with tsRNA-Ala-3–0030 inhibitor. **(I)** Western blotting was performed to evaluate ZNF70 expression in tumor tissues from xenograft nude mouse models following tsRNA-Ala-3–0030 mimic administration. *P < 0.05, **P < 0.01, ***P < 0.001.

To investigate the mechanism underlying the function of tsRNA-Ala-3-0030, we utilized TargetScan, miRanda, and RNAhybrid to predict its target genes, identifying 18 potential candidates (**Figure 4A**; **Supplementary Table S6**). Given the functional similarities between tsRNAs and miRNAs, tsRNAs are generally presumed to possess gene-silencing capabilities. To explore their potential roles in ovarian cancer, we analyzed the expression of 18 candidate target genes using TCGA data. Among these, 9 genes were upregulated, 7 were downregulated, and 2 showed no significant change (**Supplementary Figure S2-S5**). Notably, the upregulation of potential target genes appears inconsistent with the canonical gene-silencing function of tsRNAs. The data indicate that tsRNA-mediated regulation potentially operates through mechanisms that transcend immediate mRNA destabilization or translation blockade, possibly engaging indirect pathways or network-based modulatory effects. Focusing on the 4 downregulated genes, survival analysis revealed that low expression of ZNF70, ATP11C, COLGALT2, and ZNF784 was significantly associated with poor patient prognosis (**Supplementary Figure S3**). This correlation supports the hypothesis that these particular genes may be functionally relevant downstream targets of tsRNAs in ovarian cancer, where their silencing could contribute to disease progression. In order to further verify, in HEY cells transfected with tsRNA-Ala-3–0030 mimics, only ZNF70 mRNA was significantly reduced (**Figure 4B**). Experimental validation using Western blot analysis showed that overexpression of tsRNA-Ala-3–0030 mimics in HEY and OVCAR-8 cells led to a pronounced downregulation of ZNF70 protein. These results align with the proposed model in which ZNF70 functions as a target gene of tsRNA-Ala-3-0030 (**Figure 4C**). Conversely, in HEY and OVCAR-8 cells transfected with tsRNA-Ala-3–0030 inhibitors, ZNF70 protein levels were significantly upregulated (**Figure 4C**). RNAhybrid analysis predicted a binding site for tsRNA-Ala-3–0030 within the 3′-UTR of ZNF70 (**Figure 4D**), and dual-luciferase assays confirmed direct binding, as tsRNA-Ala-3–0030 significantly suppressed the luciferase activity of the ZNF70 3’UTR reporter construct (**Figure 4E**). Furthermore, ZNF70 expression was significantly reduced in ovarian cancer tissues (**Figures 4F, G**), and its expression negatively correlated with the levels of tsRNA-Ala-3–0030 in these tissues. *In vivo*, intratumoral administration of tsRNA-Ala-3–0030 antagomir resulted in elevated ZNF70 protein levels in xenograft tumors (**Figure 4H**), whereas treatment with the tsRNA-Ala-3–0030 agomir suppressed ZNF70 expression (**Figure 4I**).

**Figure 5:**
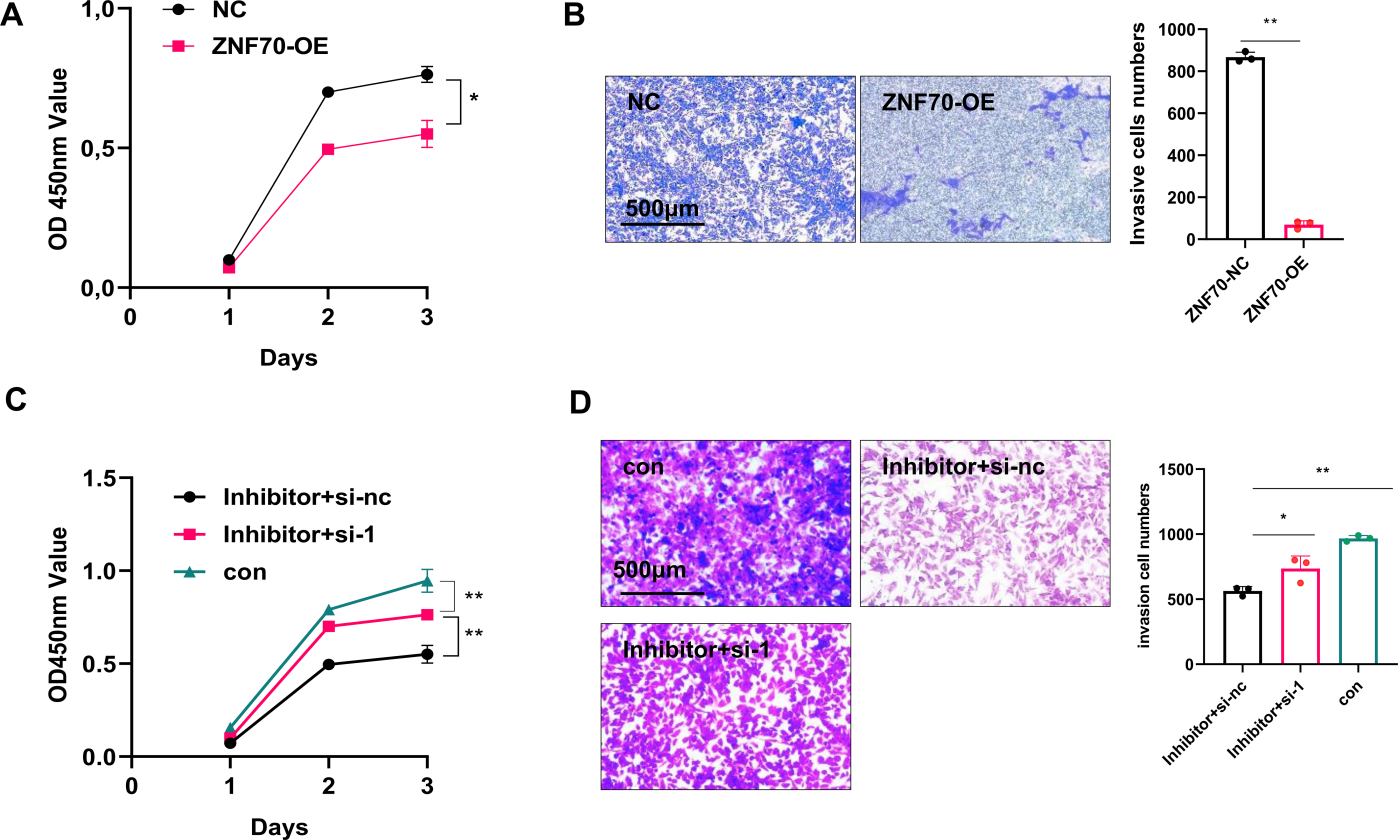
tsRNA-Ala-3–0030 modulates the function of ovarian cancer cells in a ZNF70-dependent manner. **(A)** CCK8 analysis for the growth of HEY cells transfected with ZNF70-NC or ZNF70-OE. **(B)** Transwell assays for the invasion of HEY cells transfected with ZNF70-NC or ZNF70-OE. **(C)** CCK-8 assay was performed to assess the growth of HEY cells after co-transfection with tsRNA-Ala-3–0030 inhibitor and either si-ZNF70 or si-NC. **(D)** Cell invasion was evaluated by Transwell assay in HEY cells following co-transfection with tsRNA-Ala-3–0030 inhibitor and either si-ZNF70 or si-NC. *P < 0.05, **P < 0.01.

As a Krüppel C2H2-type zinc-finger protein, ZNF70 possesses DNA-binding capacity and mediates transcriptional regulation. Overexpression of ZNF70 in HEY cells significantly inhibited cell proliferation and invasion (**Figures 5A, B**). Conversely, siRNA-mediated knockdown of ZNF70 restored proliferation and invasion in cells treated with the tsRNA-Ala-3–0030 inhibitor (**Figures 5C, D**).

Together, these findings demonstrate that tsRNA-Ala-3–0030 promotes ovarian cancer progression by suppressing ZNF70.

## Discussion

Advancements in ovarian cancer management notwithstanding, the pronounced histological and molecular heterogeneity inherent to this disease—together with interpatient variability in therapeutic response—substantially impedes the development of personalized prognostic and treatment strategies (9). Annual global diagnoses of ovarian cancer exceed 300,000 cases. The associated mortality is substantial, with roughly 50% of patients failing to survive beyond five years post-diagnosis (12). A deeper insight into the molecular mechanisms underlying ovarian cancer is crucial to address this limitation and to facilitate the advancement of targeted therapeutic strategies. In this context, tsRNAs (discovered around 2012) represent an emerging group of small RNAs that, much like microRNAs, employ Argonaute-mediated mechanisms to regulate the expression of downstream genes. tsRNAs first recognize target genes via complementary base pairing, and subsequently recruit Argonaute proteins to form the RNA-induced silencing complex (RISC), which then mediates downstream gene regulatory functions (13). In this study, we identified tsRNA-Ala-3–0030 as a novel oncogenic small RNA in ovarian cancer. Our data collectively demonstrate that the highly expressed tsRNA-Ala-3–0030 functions as a key oncogenic driver in ovarian cancer, as evidenced by its promotion of proliferation, migration, invasion, and tumor growth in experimental models. At the molecular level, tsRNA-Ala-3-0030 directly binds to and downregulates ZNF70, leading to enhanced tumor growth and progression. This mechanistic link was further substantiated by the observation that ZNF70 re-expression abrogated the oncogenic outcomes, underscoring the pathophysiological relevance of this regulatory axis in ovarian cancer.

Our findings align with recent reports highlighting the oncogenic functions of tsRNAs in diverse cancers, including ovarian cancer. Research by Panoutsopoulou et al. revealed that a specific internal tRF generated from tRNA-Gly-GCC is significantly associated with adverse prognosis and acquired chemotherapeutic resistance in ovarian cancer (14). Correspondingly, 3’U-tRF-Val-CAC facilitates ovarian cancer progression by augmenting both migratory and proliferative behaviors of cancer cells (15). The identification of tsRNA-Ala-3-0030 as an oncogenic driver further substantiates the emerging paradigm that tsRNAs, akin to miRNAs, participate in post-transcriptional gene regulation and contribute to tumorigenic processes.

Collectively, these findings define the tsRNA-Ala-3-0030/ZNF70 axis as an essential driver of ovarian carcinogenesis, which in turn exposes a novel targetable pathway for potential therapeutic intervention. This axis represents a newly defined, mechanistically grounded point of intervention. Specifically, the oncogenic activity of tsRNA-Ala-3–0030 presents a direct target for inhibition. This could be achieved through the design of small molecule inhibitors that disrupt its biogenesis, stability, or interaction with downstream effector proteins such as ZNF70. Conversely, the tumor-suppressive function of ZNF70, which is dampened by tsRNA-Ala-3-0030, offers a complementary strategy. Therapeutic efforts could focus on developing ZNF70 activators or stabilizers—for instance, molecules that enhance its transcription, prevent its ubiquitin-mediated degradation, or mimic its functional activity—to restore its normal regulatory role in counteracting tumor growth and invasion. Targeting this axis offers a dual-pronged, precision-based approach: silencing a pro-tumorigenic non-coding RNA while simultaneously reactivating a compromised tumor suppressor pathway, which together could form the basis of a viable and potent therapeutic strategy for ovarian cancer. Clinically, tsRNA-Ala-3-0030 exhibits a statistically significant expression disparity between FIGO stage II and stages III–IV ovarian cancer cases, as evidenced by our preliminary data. Such stage-associated expression supports its prospective role as a biomarker for assessing pathological progression and staging accuracy.

Functionally, tsRNAs often participate in Argonaute-facilitated post-transcriptional silencing, similar to miRNAs, through sequence-specific interactions with 3’-UTR sites of messenger RNAs. Our study demonstrated that tsRNA-Ala-3–0030 binds directly to the 3′-UTR of ZNF70 and suppresses its expression. ZNF70, a KRAB zinc-finger protein, mediates transcriptional repression by recruiting or cooperating with chromatin-remodeling complexes (16–18). Previous studies have elucidated the primary mechanism of transcriptional repression mediated by KRAB-ZFPs. In this process, the C-terminal C_2_H_2_ zinc finger domains of KRAB-ZFPs specifically recognize and bind to target DNA sequences, while the N-terminal KRAB domains recruit KAP1 (TRIM28). Once bound, KAP1 serves as a scaffold for the assembly of a multiprotein repressor complex by interacting with several key chromatin-modifying factors, including the NuRD/HDAC complexes, the histone methyltransferase SETDB1, DNA methyltransferases (DNMTs), and heterochromatin protein 1 (HP1). As a result of this integrated recruitment, chromatin adopts a compacted state at specific loci, leading to their stable epigenetic repression (19–21). Downregulation of ZNF70 has been implicated in gastric cancer and esophageal squamous cell carcinoma, but its role in ovarian cancer had not been previously described (16, 17). Our data provide the first evidence that ZNF70 acts as a tumor suppressor in OC and that its inhibition by tsRNA-Ala-3–0030 contributes to malignant progression.

In light of the unfavorable prognosis associated with ovarian cancer and the pressing demand for new biomarkers, tsRNA-Ala-3-0030 carries considerable clinical relevance. Its consistent overexpression in tumor tissues, coupled with an inverse relationship to ZNF70 expression, indicates its viability as both a diagnostic and prognostic indicator. Moreover, *in vivo* studies demonstrate that inhibiting tsRNA-Ala-3-0030 not only rescues ZNF70 expression but also curbs tumor growth, underscoring its potential as a therapeutic target. With the emergence of RNA-based therapeutics, antagomirs targeting oncogenic tsRNAs may provide a novel strategy for precision oncology in ovarian cancer.

Several limitations should be acknowledged. The study is constrained by the relatively small clinical tissue sample set, underscoring the need for validation in larger populations to determine the prognostic utility of tsRNA-Ala-3-0030. Furthermore, while ZNF70 has been verified as a direct target, the possibility that tsRNA-Ala-3-0030 influences additional genes or signaling networks cannot be excluded and warrants systematic investigation. Subsequent work should also evaluate the presence of tsRNA-Ala-3-0030 in circulating biofluids (e.g., serum or exosomes), a step critical to establishing its viability as a non-invasive biomarker. ZNF70’s potential functions within signaling pathways or regulatory networks hold significant research value, warranting further in-depth investigation. Finally, preclinical evaluation of tsRNA-targeting therapies in combination with standard chemotherapy may provide further insight into its therapeutic potential.

Taken together, our findings reveal tsRNA-Ala-3-0030 as an ovarian cancer-associated tsRNA that enhances tumorigenesis by inhibiting ZNF70 expression. These observations extend the current knowledge of tsRNA mechanisms and nominate tsRNA-Ala-3-0030 for biomarker and therapeutic development. Ongoing research into tsRNA-mediated gene networks will further clarify the pathophysiology of ovarian cancer and support the design of targeted interventions.

## Data Availability

The datasets presented in this study can be found in online repositories. The names of the repository/repositories and accession number(s) can be found in the article/**Supplementary Material**.
