# Supplementary material for: tsRNA-Ala-3–0030 drives ovarian cancer progression by suppressing ZNF70
Source: Front Oncol. 2026 Jan 29;16:1738006. doi: 10.3389/fonc.2026.1738006 (PMC12894024; doi:10.3389/fonc.2026.1738006)

**Supplement materials**

| Features | Relative expression of tsRNA-Ala-3-0030 | | |
| --- | --- | --- | --- |
| Mean Age (± SD) | 53.46 ± 7.75 years | | |
| Age Range | 40 - 71 years | | |
| FIGO Stage Distribution | Stage IIB: 9 cases (37.5%)  Stage IIIC: 8 cases (33.3%)  Stage IIIA1: 4 cases (16.7%)  Stage IVB: 3 cases (12.5%) | | |
|  | Sample Size | Median log₁₀(J) (IQR) | *p*-value |
| Early-stage Group (IIB) | 9 | 0.78 (-0.08 – 1.40) | 0.022 |
| Advanced-stage Group (IIIA1+IIIC+IVB) | 15 | 1.23 (0.80 – 1.61) |  |

**Table S1. Patient demographics.**

**Table S2. Statistical Description of Differentially Expressed Genes in the TCGA-OV Cohort .**

| Gene | Group | n | Median (IQR) | Mean (± SD) | Range  (Min - Max) |
| --- | --- | --- | --- | --- | --- |
| ZNF70 | Normal | 88 | 2.046 (1.890–2.289) | 2.057 ± 0.317 | 0.536 – 2.595 |
|  | Tumor | 427 | 0.978 (0.660–1.319) | 1.019 ± 0.496 | 0.000 – 5.051 |
| ATP11C | Normal | 88 | 3.806 (3.690–3.976) | 3.793 ± 0.294 | 2.570 – 4.442 |
|  | Tumor | 427 | 2.378 (1.926–2.859) | 2.394 ± 0.695 | 0.000 – 4.870 |
| COLGALT2 | Normal | 88 | 0.891 (0.541–1.184) | 0.997 ± 0.695 | 0.138 – 3.914 |
|  | Tumor | 427 | 0.333 (0.176–0.609) | 0.502 ± 0.493 | 0.000 – 2.872 |
| UBE2V2 | Normal | 88 | 4.373 (4.210–4.525) | 4.400 ± 0.350 | 3.614 – 5.447 |
|  | Tumor | 427 | 4.921 (4.495–5.316) | 4.872 ± 0.652 | 0.000 – 6.658 |
| CALCR | Normal | 88 | 0.000 (0.000–0.018) | 0.017 ± 0.027 | 0.000 – 0.111 |
|  | Tumor | 427 | 0.043 (0.014–0.098) | 0.073 ± 0.126 | 0.000 – 1.811 |
| DUOXA1 | Normal | 88 | 0.993 (0.683–1.338) | 1.097 ± 0.818 | 0.057 – 7.043 |
|  | Tumor | 427 | 0.642 (0.299–1.227) | 0.882 ± 0.794 | 0.000 – 5.089 |
| FAM3B | Normal | 88 | 0.202 (0.098–0.370) | 0.300 ± 0.480 | 0.000 – 4.268 |
|  | Tumor | 427 | 3.111 (2.044–4.385) | 3.196 ± 1.535 | 0.000 – 6.924 |
| CHURC1 | Normal | 88 | 4.970 (4.829–5.132) | 4.968 ± 0.249 | 4.076 – 5.660 |
|  | Tumor | 427 | 5.309 (4.949–5.667) | 5.296 ± 0.627 | 0.000 – 6.821 |
| TRA2B | Normal | 88 | 6.626 (6.515–6.810) | 6.637 ± 0.260 | 5.476 – 7.380 |
|  | Tumor | 427 | 6.676 (6.345–7.037) | 6.640 ± 0.657 | 0.000 – 9.314 |
| RNF157 | Normal | 88 | 1.111 (0.826–1.435) | 1.219 ± 0.541 | 0.310 – 2.778 |
|  | Tumor | 427 | 1.195 (0.722–1.868) | 1.416 ± 0.918 | 0.000 – 4.836 |
| WDR5B | Normal | 88 | 1.811 (1.540–2.056) | 1.793 ± 0.380 | 0.705 – 2.599 |
|  | Tumor | 427 | 2.587 (2.197–2.957) | 2.582 ± 0.572 | 0.000 – 4.224 |
| EFNB2 | Normal | 88 | 2.425 (1.767–3.205) | 2.586 ± 1.019 | 0.895 – 5.409 |
|  | Tumor | 427 | 3.834 (3.042–4.666) | 3.809 ± 1.155 | 0.000 – 7.305 |
| ZNF784 | Normal | 88 | 3.659 (3.372–3.940) | 3.646 ± 0.410 | 2.284 – 4.513 |
|  | Tumor | 427 | 2.513 (2.109–2.923) | 2.510 ± 0.620 | 0.000 – 4.179 |
| CAPN15 | Normal | 88 | 4.496 (4.247–4.795) | 4.494 ± 0.397 | 3.233 – 5.798 |
|  | Tumor | 427 | 4.427 (3.945–4.855) | 4.379 ± 0.722 | 0.000 – 6.277 |
| MYO5A | Normal | 88 | 2.527 (2.318–2.724) | 2.530 ± 0.323 | 1.170 – 3.483 |
|  | Tumor | 427 | 1.816 (1.278–2.349) | 1.836 ± 0.721 | 0.000 – 4.503 |
| HUNK | Normal | 88 | 0.470 (0.214–0.725) | 0.533 ± 0.392 | 0.029 – 1.714 |
|  | Tumor | 427 | 2.236 (1.606–3.017) | 2.304 ± 0.992 | 0.000 – 5.046 |
| TAF8 | Normal | 88 | 3.083 (2.898–3.193) | 3.060 ± 0.297 | 2.018 – 3.719 |
|  | Tumor | 427 | 3.183 (2.786–3.657) | 3.210 ± 0.696 | 0.000 – 7.327 |
| GTDC1 | Normal | 88 | 3.690 (3.534–3.888) | 3.693 ± 0.316 | 2.727 – 4.654 |
|  | Tumor | 427 | 3.048 (2.574–3.478) | 3.001 ± 0.660 | 0.000 – 4.519 |

**Table footnote:**
Expression values are presented as log₂-transformed transcripts per million (TPM). Core statistical descriptors include sample size (n), median with interquartile range (IQR), mean with standard deviation (SD), and range (minimum–maximum). Genes are listed in alphabetical order for quick reference.

**Table S3. The sequences of TsRNA mimics and inhibitors.**

| gene | Sequence(5’-3’) | Sequence(3’-5’) |
| --- | --- | --- |
| tsRNA-Ala-3-0030 mimic (agomir) | GUUCGAUCCCCAGCAUCUCCACCA | UGGUGGAGAUGCUGGGGAUCGAAC |
| tsRNA-Ala-3-0030 inhibitor (antagomir) | UGGUGGAGAUGCUGGGGAUCGAAC |  |
| NC mimic(agomir) | UUUGUACUACACAAAAGUACUG | CAGUACUUUUGUGUAGUACAAA |
| NCinhibitor(antagomir) | UUUGUACUACACAAAAGUACUG |  |

**Table S4. The sequence of small interfering RNAs targeting human ZNF70.**

| gene | Sense(5’-3’) | Antisense(5’-3’) |
| --- | --- | --- |
| NC siRNA | UUCUCCGAACGUGUCACGUTT | ACGUGACACGUUCGGAGAATT |
| ZNF70 siRNA-592 | GGAAAGAGGUUUGGAGCAATT | UUGCUCCAAACCUCUUUCCTT |
| ZNF70 siRNA-1240 | GUGUAAGGAAUGUGGGAAATT | UUUCCCACAUUCCUUACACTT |
| ZNF70 siRNA-1391 | GGGAAGAAACCAUACAAAUTT | AUUUGUAUGGUUUCUUCCCTT |

**Table S5. The primer sequences of RT-qPCR.**

| gene | reverse transcription primer(5’-3’) | Forward(5’-3’) | Reverse(5’-3’) |
| --- | --- | --- | --- |
| tsRNA-Ala-3-0030 | GTCGTATCCAGTGCAGGGTCCGAGGTATTCGCACTGGATACGACTGGTGG | GGTTCGATCCCCAGCATCT | AGTGCAGGGTCCGAGGTATT |
| tsRNA-Gly-5-0010 | GTCGTATCCAGTGCAGGGTCCGAGGTATTCGCACTGGATACGACACCACT | GCGGCGCCGCTGGTGT | AGTGCAGGGTCCGAGGTATT |
| TsRNA-Glu-5-0010 | GTCGTATCCAGTGCAGGGTCCGAGGTATTCGCACTGGATACGACACTAGA | CGCGCGTCCGTGGTGG | AGTGCAGGGTCCGAGGTATT |
| U6 | / | CTCGCTTCGGCAGCACA | AACGCTTCACGAATTTGCGT |
| β-actin |  | CTCCATCCTGGCCTCGCTGT | GCTGTCACCTTCACCGTTTT |
| GAPDH | / | GTCTCCTCTGACTTCAACAGCG | ACCACCCTGTTGCTGTAGCCAA |
| ZNF70 | / | AACAGGTTAGAGTCACAACAAGG | CACAAACTGAAATTCCCCTCGTA |
| ATP11C | / | AGTACGTGATACCATTGCACTG | CGTAGCTCCTTTCAGCAAGAGA |
| COLGALT2 | / | CAATGTGGCAAACCTGGTCG | ACTTGAGAGGAGGACGGGAT |
| ZNF784 | / | GCCAGGTTCTTTCCACTGTG | CCCCGTGTGCAAGCTGTAG |

**Table S6. The target of tsRNA-Ala-3-0030 in TargetScan, miRanda, and RNAhybrid.**

| Seqname | GeneSymbol | Type | Context+ | Context | Structure |
| --- | --- | --- | --- | --- | --- |
| NM_000259 | MYO5A | Coding | -0.067 | -0.081 | 160 |
| NM_001204064 | CHURC1 | Coding | -0.221 | -0.205 | 150 |
| NM_001243879 | TRA2B | Coding | -0.221 | -0.183 | 304 |
| NM_001276266 | DUOXA1 | Coding | -0.286 | -0.146 | 167 |
| NM_001284233 | GTDC1 | Coding | -0.003 | 0.124 | 444 |
| NM_001303421 | COLGALT2 | Coding | -0.496 | -0.486 | 281 |
| NM_001330501 | RNF157 | Coding | -0.209 | -0.206 | 291 |
| NM_001353812 | ATP11C | Coding | -0.223 | -0.202 | 151 |
| NM_001372056 | EFNB2 | Coding | -0.142 | -0.142 | 159 |
| NM_001742 | CALCR | Coding | -0.344 | -0.248 | 156 |
| NM_003350 | UBE2V2 | Coding | -0.387 | -0.289 | 158 |
| NM_005632 | CAPN15 | Coding | -0.077 | -0.027 | 158 |
| NM_014586 | HUNK | Coding | -0.036 | -0.021 | 287 |
| NM_019069 | WDR5B | Coding | -0.151 | -0.15 | 167 |
| NM_021916 | ZNF70 | Coding | -0.395 | -0.277 | 325 |
| NM_058186 | FAM3B | Coding | -0.229 | -0.189 | 156 |
| NM_138572 | TAF8 | Coding | -0.033 | -0.046 | 283 |
| NM_203374 | ZNF784 | Coding | -0.125 | -0.07 | 160 |

Seqname: the name of the sequence.

GeneSymbol: the official gene symbol of the sequence.

Type: the type of the transcript.

Context+: the sum of the context+ scores used in TargetScan after version 6.0, more negative is better.

Context: the sum of the context scores used in TargetScan before version 5.x, more negative is better. Structure: the sum of the structure scores used in miRanda, the higher is better.

**Figure S1. (A, B)** qPCR analysis of the expression of tsRNA-GLU-5-0010 and tsRNA-GLY-5-0010 in ovarian cancer tissues and corresponding normal tissues. **(C)** The quantitative analysis of Ki-67 staining of the tumor tissues from the xenograft tumor nude mice models treated with tsRNA-Ala-3-0030 Mimic or inhibitor. **(D-E)** qPCR and western blot analysis of ZNF70 expression in HEY cells transfected with different ZNF70 siRNAs. **P* < 0.05, ** *P* < 0.01.





**Figure S2.** The upregulated gene in ovarian cancers according to the TCGA database. **P* < 0.05, ****P* < 0.001.

**

**

**Figure S3.** The downregulated gene in ovarian cancers according to the TCGA database. ****P* < 0.001.





**Figure S4.** The downregulated gene in ovarian cancers according to the TCGA database. ***P* < 0.01,****P* < 0.001.





**Figure S5.** The unchanged gene in ovarian cancers according to the TCGA database.


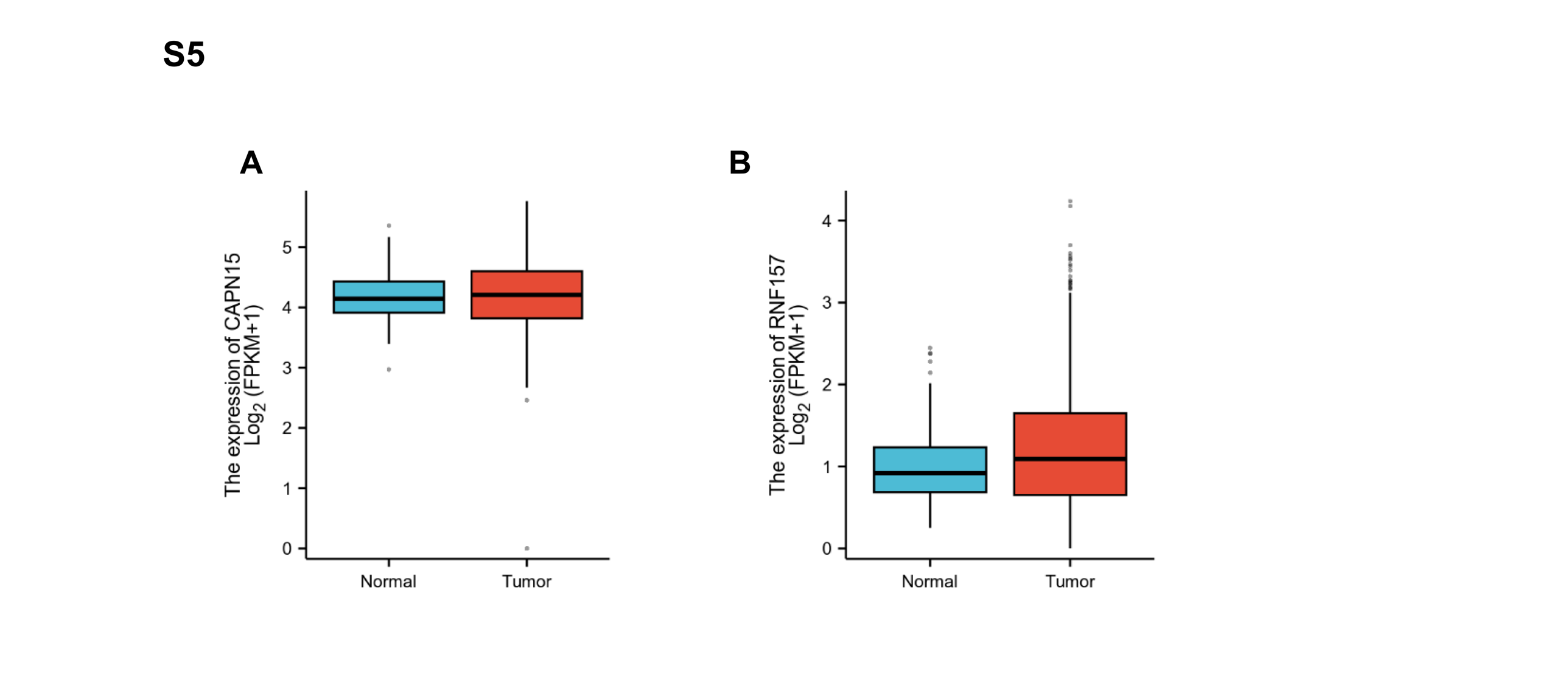


**Figure S6. A** ZNF70 protein expression is negative in this mucinous cystadenocarcinoma (Patient 3323). **B** In this serous cystadenocarcinoma (Patient 3146), immunohistochemical staining shows low positivity with moderate intensity. **C** In this serous cystadenocarcinoma (Patient 2082), immunohistochemical staining demonstrates medium positivity with moderate intensity, localized to the cytoplasmic membrane. These figures are adapted from the Human Protein Atlas database ([https://www.proteinatlas.org](https://www.proteinatlas.org/)), with reference to: Uhlen M, et al. Science. 2015; 347(6220):1260419. These images are used under the terms of the Creative Commons Attribution 4.0 International License.


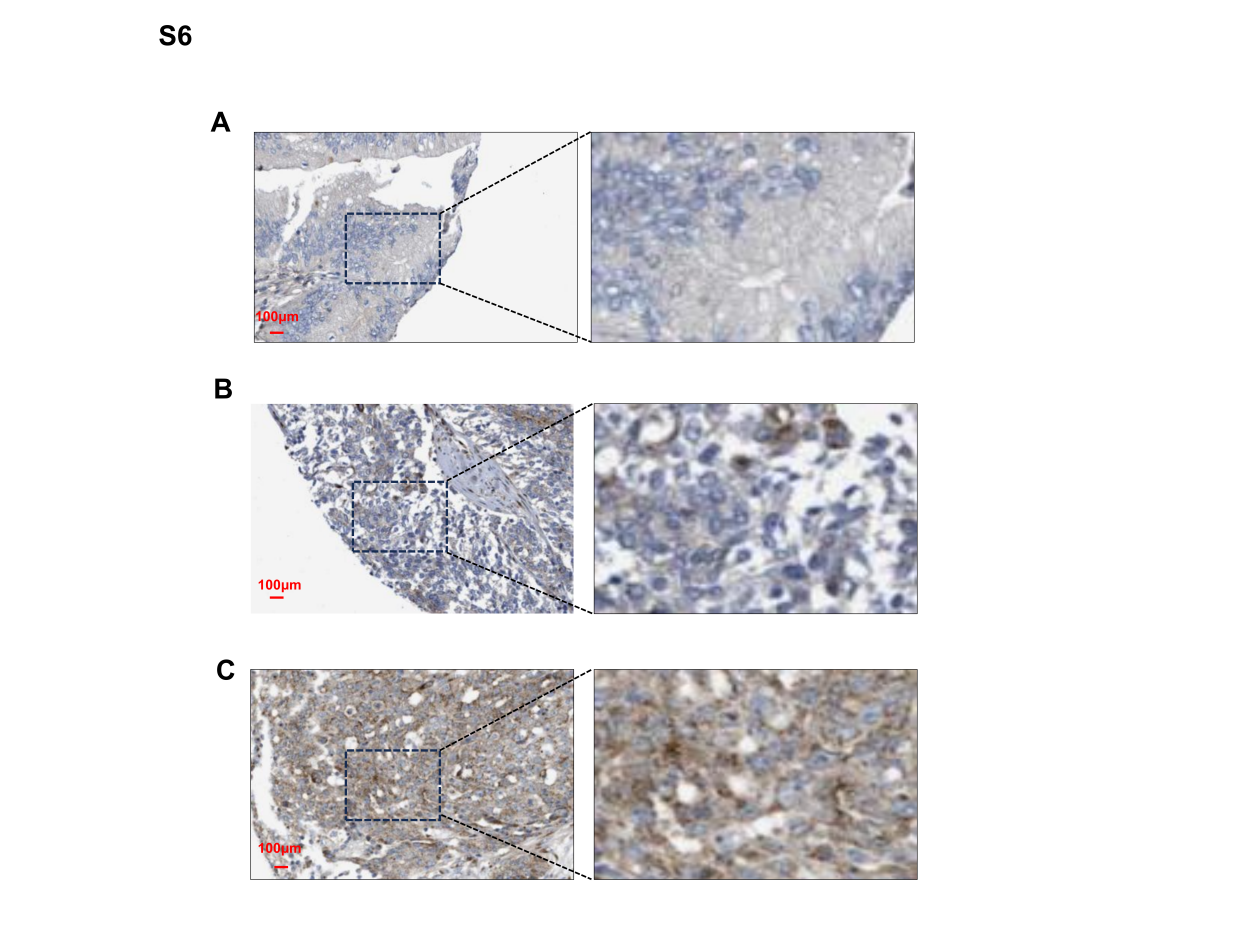


**Figure S7. The raw data of western blotting.**

**
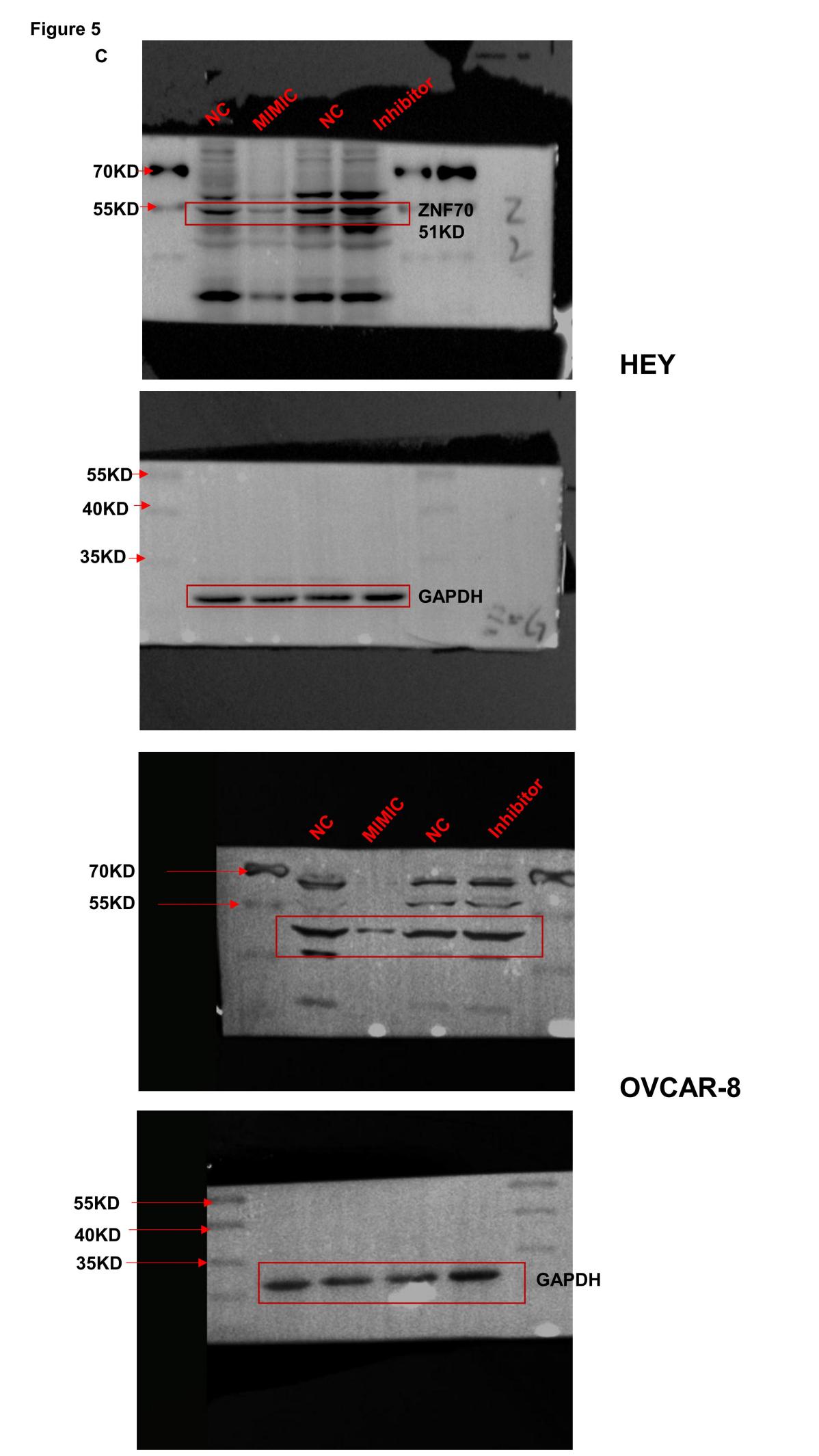

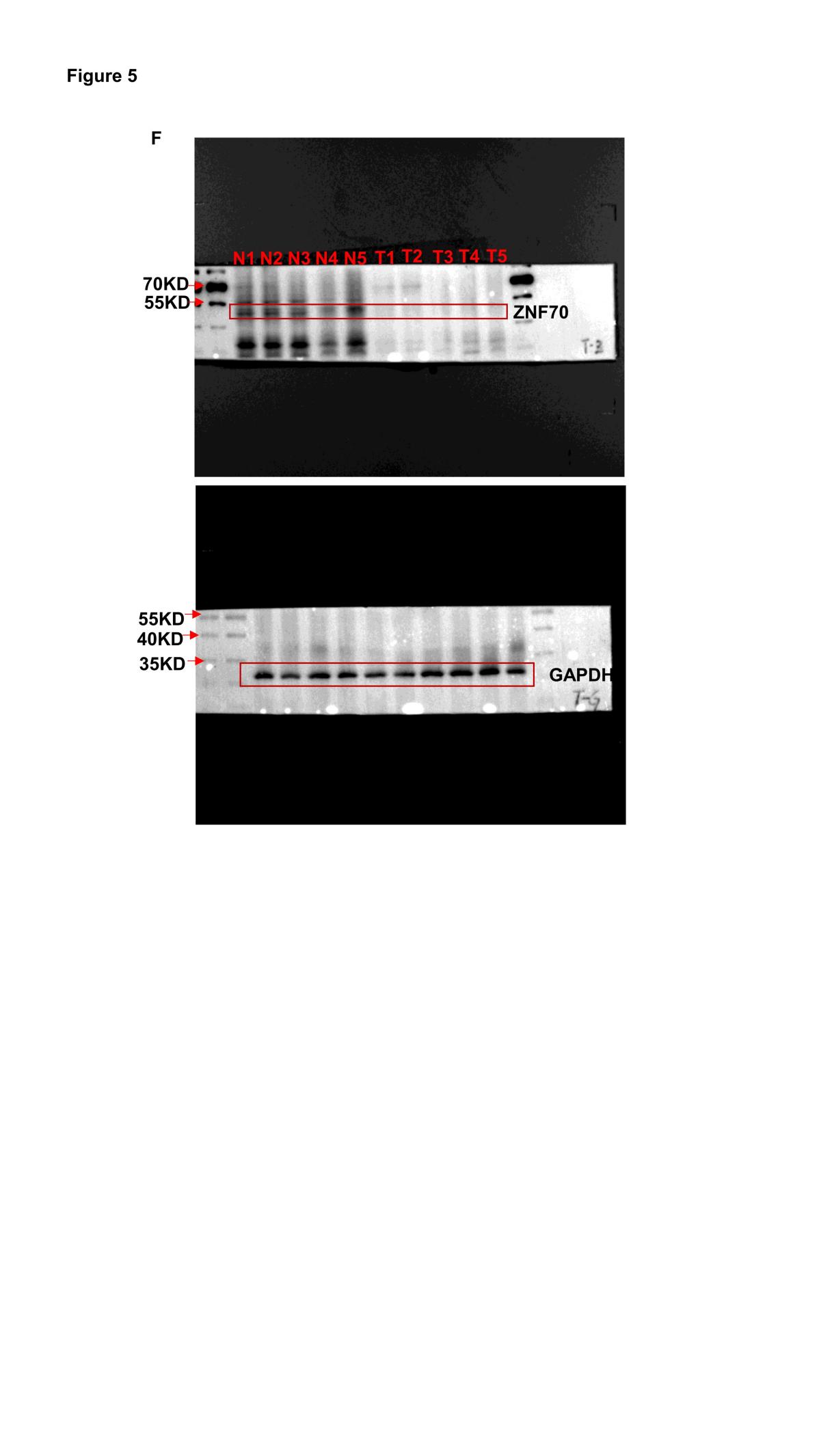
**


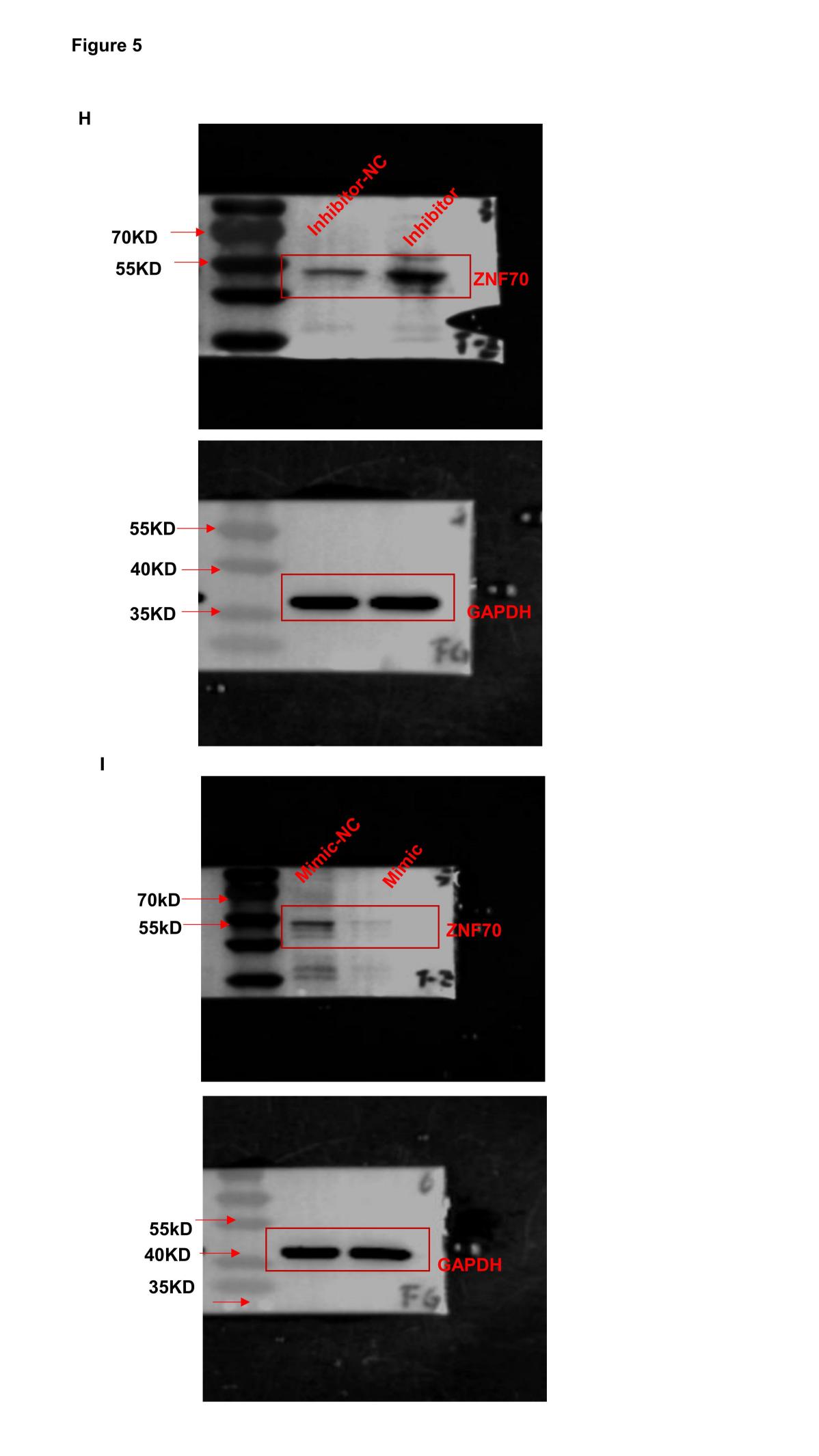

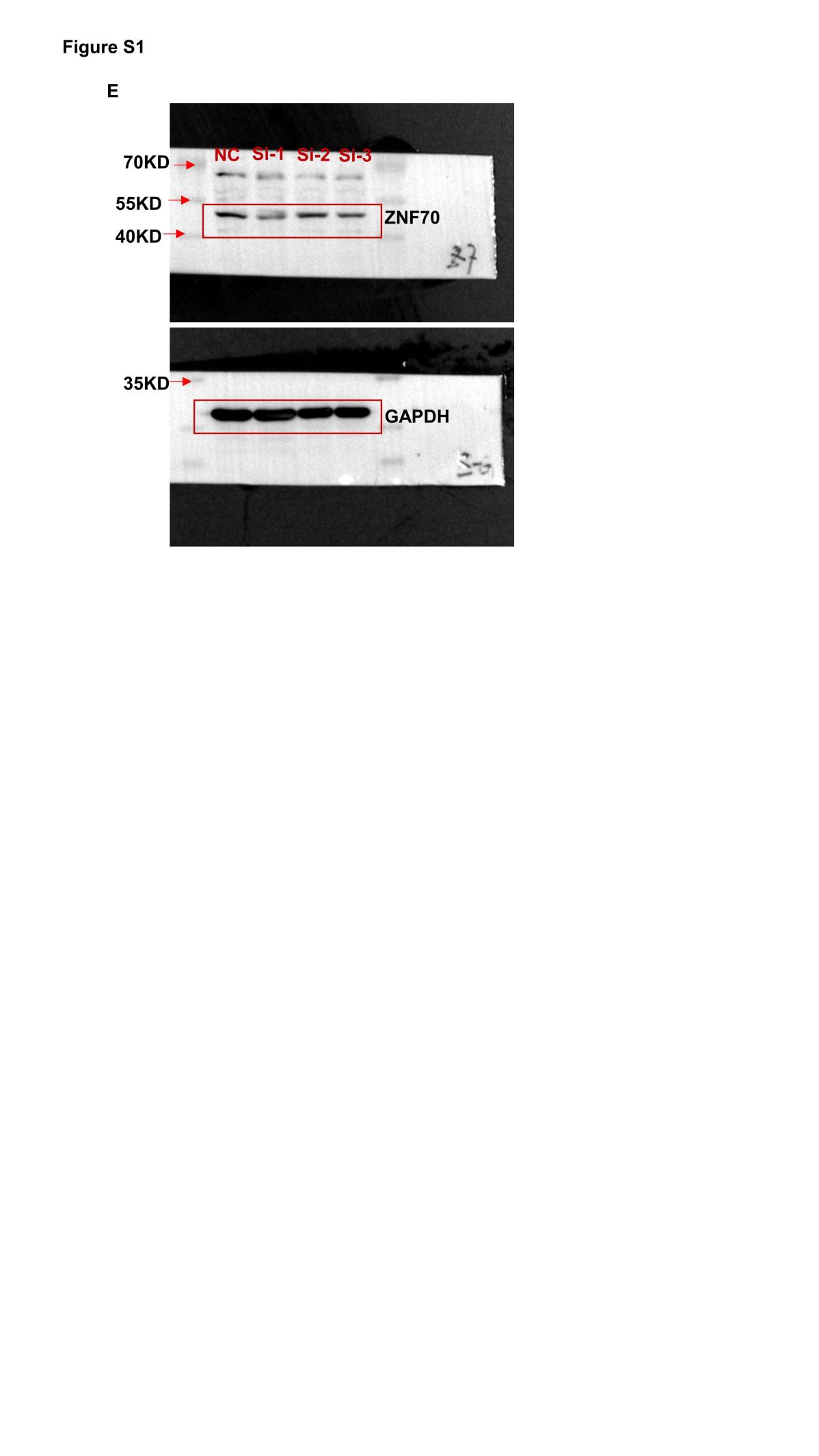

Supplement: Supplementary file 1 [file DataSheet1.docx]
